# The transcatheter arterial chemoembolization combined with targeted nanoparticle delivering sorafenib system for the treatment of microvascular invasion of hepatocellular carcinoma

**DOI:** 10.1080/21655979.2021.2001239

**Published:** 2021-12-19

**Authors:** Dongna Su

**Affiliations:** Department of Infectious Diseases, The Second Clinical Medical College (Shenzhen People’s Hospital), Jinan University, Shenzhen, P.R. China

**Keywords:** Ab-SFB-NP, transcatheter arterial chemoembolization, hepatocellular carcinoma, glypican-3, tumor control rate

## Abstract

to explore the value of transcatheter arterial chemoembolization (TACE) combined with targeted nanoparticle delivery system for sorafenib (SFB) to treat hepatocellular carcinoma (HCC) with microvascular invasion. 42 HCC patients with microvascular invasion after liver cancer surgery were selected from our hospital from December 2020 and February 2021. Patients were divided into experimental group and control group based on their willingness. Patients in experimental group (18 cases) were treated with combination therapy of TACE and Ab-SFB-NP system; while patients in control group (24 cases) took TACE and non-nano drug delivery system. There was no obvious difference in liver function and blood test results between two groups of patients before treatment and one month after treatment (*P* > 0.05). Three months after treatment, differences of alanine aminotransferase (ALT) were statistically significant (*P* < 0.05); while differences of other test results were not (*P* > 0.05). The disease control rate (DCR) of patients in experimental group was higher slightly (*P* > 0.05). The incidence of adverse reactions of patients in experimental group was lower than the control group and the differences were statistically significant (*P* < 0.05). After three months of TACE, the DCR in the experimental group was significantly higher compared to control group. The toxic reactions of taking SFB with Ab-SFB-NP nano-drug delivery system mainly included hand-foot syndrome, diarrhea, and bleeding, the toxic reactions were mainly at level 1 ~ 2. After symptomatic treatment, the toxicity was effectively controlled, so the security was high.

## 1. Introduction

Primary liver cancer, referred to as liver cancer. According to statistics, the fatality rate of liver cancer in China ranks third, and the number of patients suffering from it accounts for about 50% of the world [1]. The prevalence of men is about 3.5 times that of women [2], and the main cause is chronic hepatitis B virus infection [3]. Hepatocellular carcinoma (HCC) is the most common [4]. Microvascular invasion (MVI) is a high-signal indication of postoperative recurrence of liver cancer, which is mainly manifested by the appearance of cancerous masses in the vascular cavity covered by endothelial cells under the microscope, and MVI mostly occurs on the small branches of the portal vein inside the liver tissue around the lesion. MVI suggests poor prognosis of liver cancer and aggressive behavior of cancer cells [5]. So far, the first choice for the treatment of liver cancer is still surgical resection, but more than 90% of patients have been ineffective when they are diagnosed. Although conventional chemotherapy is effective, it can have serious side effects.

At this stage, transcatheter arterial chemoembolization (TACE) is recognized as the first-line non-surgical treatment for liver cancer [6]. TACE refers to the use of embolic agents to block tumor blood vessels, causing necrosis and apoptosis of cancer cells. It is not only less traumatic, but it can also be reused. It also has the characteristics of targeting and is easily accepted by patients [7]. However, this method still has some shortcomings, such as residual lesions and proliferation. Studies showed that under the influence of certain factors, the activity of vascular endothelial growth factor was enhanced, which promotes the formation of blood vessels and causes the recurrence and metastasis of cancer [8].

Another method is for the treatment of Raf kinase in the development of HCC. Sorafenib (SFB) is a systemic treatment drug. It has been proven to prolong the survival of patients with advanced HCC. The drug is approved for clinical treatment [9]. However, due to the way of medication and SFB itself, the targeting effect of the drug is poor, and the clinical efficacy is limited [10]. In order to overcome this defect, experts have developed a corresponding antibody-specific anti-GPC3 monoclonal (Anti-GPC3 Ab) through the relatively specific expression molecule glypican-3 (GPC3) of liver cancer. It has the ability to specifically target liver cancer cell and the anti-GPC3 Ab were combined with nanoparticles (NPs), an AB-SFB-NP nano-drug delivery system with large drug loading capacity, good targeting, high safety, stable, and controllable drug release rate in the tumor was constructed [11].

In this study, it aimed to investigate the application value of transcatheter arterial chemoembolization (TACE) combined with targeted nanoparticle delivery of sorafenib (SFB) system in the treatment of hepatocellular carcinoma with microvascular invasion. If the therapeutic effect of Ab-SFB-NP nano delivery system is more prominent, it can further provide a reasonable research basis for the good application prospect of nanotechnology in the medical field.

### 1.1. Research object

42 patients with microvascular invasion of hepatocellular carcinoma after liver cancer surgery were selected as the research objects in our hospital from December 2020 and February 2021. Patients were divided into experimental group and control group based on their willingness to take SFB drug targeted for nanoparticle delivery after the operation. Patients in the experimental group were treated with the combination therapy of TACE and Ab-SFB-NP nano-drug delivery system, a total of 18 cases. The control group took TACE and non-nano drug delivery system to take SFB for treatment, a total of 24 cases. The research was complied with the *Declaration of Helsinki* and was approved by the ethics review committee.

Inclusion criteria were as follows: a. Patients with liver cancer diagnosed used the same criteria (American Association for the Study of Liver Diseases (AASLD) [12] guidelines); b. Patients were all over the age of 18; c. Patients without contraindications to chemotherapy; d. Patients with Barcelona Clinic Liver Cancer (BCLC) [13] B or C; e. Patients whose liver function was at Child-Pugh level [14] A/B; f. Patients whose life expectancy was more than 3 months.

Exclusion criteria were as follows: a. Patients with severely insufficient coagulation; b. Those who had received other treatments; c. Patients with Child-Pugh level C; d. Patients with severely inadequate functions of the heart, lungs, kidneys, and other organs.

### 1.2. Research method

**Figure 1.**
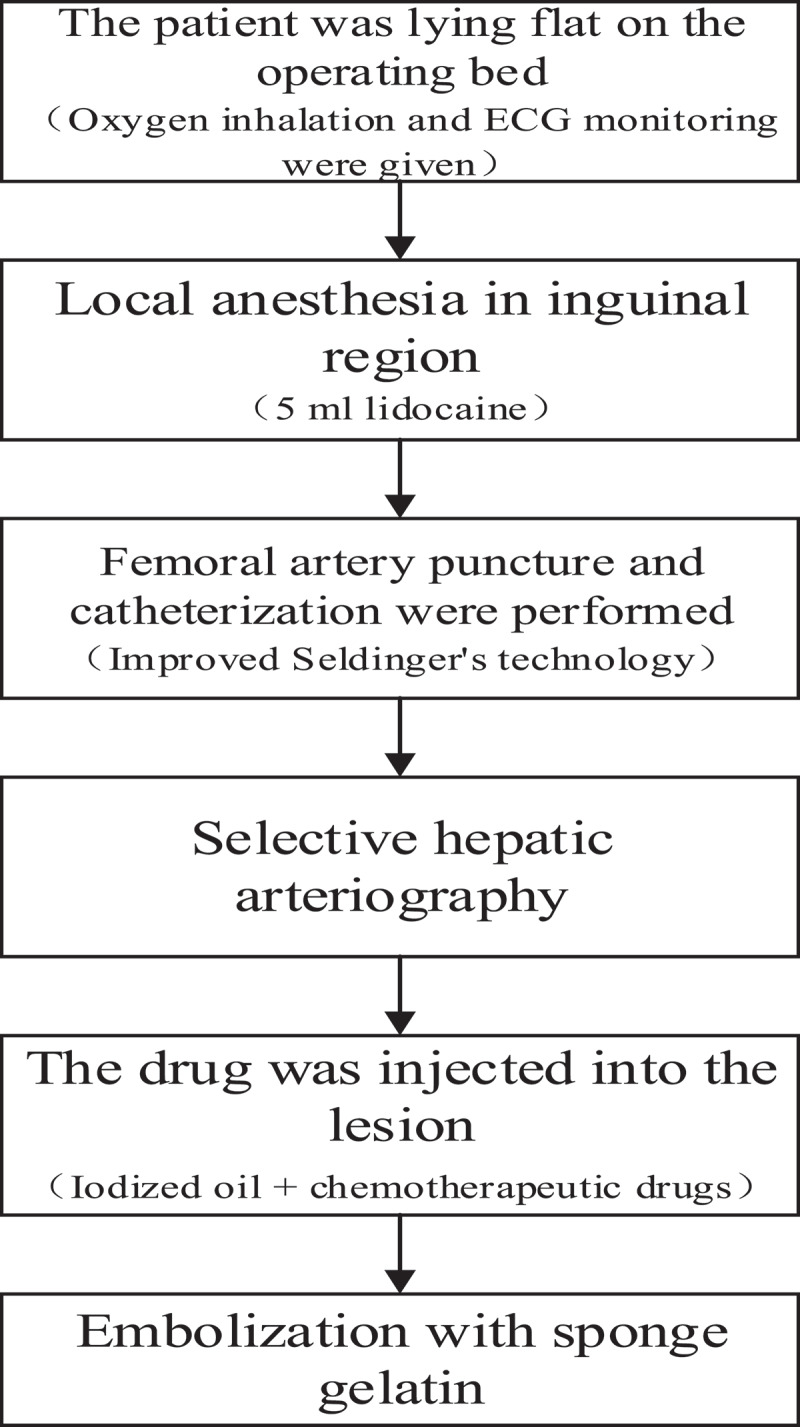
Surgical flow of TACE treatment

I. All patients received TACE treatment first, and the surgical procedure was shown in Figure 1. The control group was treated with TACE only, while the experimental group was treated with AB-SFB-NP nano-drug delivery system and non-AB-SFB-NP nano-drug delivery system on the fourth day after TACE treatment, with 21 days as a course of treatment.

**Figure 2.**
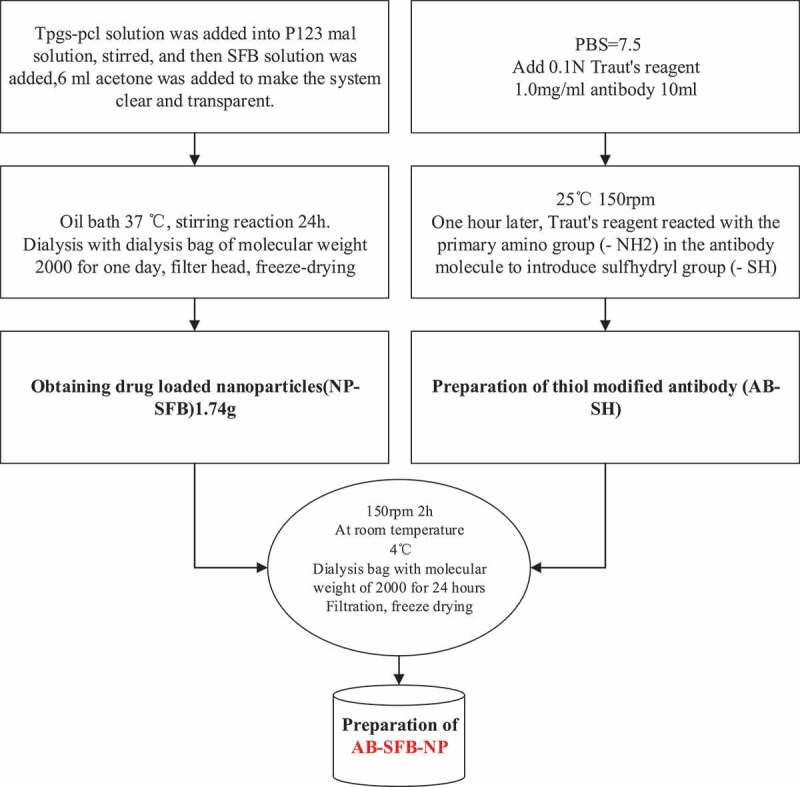
Preparation process of AB-SFB-NP drug

II. Preparation of AB-SFB-NP drugs: In this research, NP-SFB nanoparticles loaded with SFB were prepared by the improved nano-precipitation method [15] in the acetone-water system, and then the thiol-modified antibody (AB-SH) was obtained by modifying the antibody with Traut’ reagent. Then, NP-SFB and AB-SH were mixed at room temperature for two hours to obtain AB-SFB-NP. Before the preparation, 1.8 g P123-MAL, 300 mg TPGS-PCL, and 67 mg SFB were weighed, respectively. They were dissolved in 40 mL, 8 mL water, and 4 mL acetone for preparation. The specific operation process was shown in Figure 2. (The above chemical materials for the preparation of Ab-SFB-NP drugs were obtained from Alfa Aesar Chemical Co., Ltd., China, and the antibodies used were obtained from Sigma-Aldrich, USA)

III. Characterization of P123-MAL, TPGS-PCL, SFB-NPs

A. The structure was characterized by nuclear magnetic resonance analyzer (Bruker, Germany, model 1 H NMR Bruker-300), and the type of H atom was analyzed. The specific operation was as follows: 3–5 mg of sample was placed in deuterated chloroform solution to dissolve it, then loaded into a nuclear magnetic tube, and the structure was analyzed by nuclear magnetic resonance hydrogen spectroscopy (1 H NMRBruker-300).

B. The particle size and size distribution of nanoparticles were measured by nanolaser particle sizer (Malvern, UK, model Zetasizer Nano ZS), the zeta potential of nanoparticles was measured by laser Doppler microelectrophoresis, 2 mg of dried particle sample was weighed before measurement, resuspended in deionized water to prepare 1 mg/mL of suspension, and then dispersed by ultrasound. The measurements were repeated three times using the above instruments.

C. Drug loading characterization. Sorafenib solutions prepared with different concentration gradients were weighed to obtain a standard curve using high performance liquid chromatography (HPLC) [16], and then the nanomedicine samples were dissolved in dimethyl sulfoxide to decompose and destroy the nanomedicine and detect the drug load.

IV. Experimental results of AB-SFB-NP before application in human body

Studies showed that taking Ab-SFB-NP in vivo can effectively limit tumor growth. It was found through experiments that the main mechanism of AB-SFB-NP was reducing the occurrence of MEK1/2 and ERK phosphorylation by inhibiting the activity of Raf kinase, and the Raf /MEK/ERK signaling pathway was further inhibited [17]. In addition, the activity of Mcl-1 was reduced by AB-SFB-NP, the polymerization of Bax and Bak on the mitochondrial membrane was induced, and the mitochondrial permeability was increased so that the mitochondrial production and release of cytochrome C was also be increased, thus leading to cell apoptosis, and there was a synergistic effect between the two. The toxicity of AB-SFB-NP can be controlled and Ab – SFB – NP drugs can be used in clinical trials.

### 1.3. Observation indicators

I. General information including age, gender, etiology, Eastern Cooperative Oncology Group (ECOG) [18] score, BCLC stage, and Child-Pugh level were observed before enrollment of patients in the two groups.

II. Preoperative blood routine, liver function, and other laboratory indicators as well as enhanced CT or MRI results of all patients were recorded.

III. The blood routine, liver function, other laboratory indexes, and imaging results were recorded one month and three months after TACE.

IV. Adverse reactions were observed after medication.

### 1.4. Evaluation criteria of treatment effect

I. After postoperative enhanced CT/MRI detection, the efficacy was evaluated according to the actual change of tumor efficacy evaluation criteria (MRECIST) [19], as shown in Table 1.

**Table 1.** MRECIST evaluation criteria

The development of the condition	Complete response (CR)	Partial relief (PR)	Progression of disease (PD)	Stable disease (SD)
Standard	Arterial enhancement disappeared in all target lesions	The total diameter of the target lesion arterial phase enhanced imaging was reduced by at least 30%;	The total diameter of the target lesions during the arterial phase enhancement increased by more than 20%, or new lesions appeared	The diameter increases and decreases of the target lesion was between PR and PD

Tumor control rate (DCR) = (CR+PR+SD/total number of cases X 100%).

**Figure 3.**
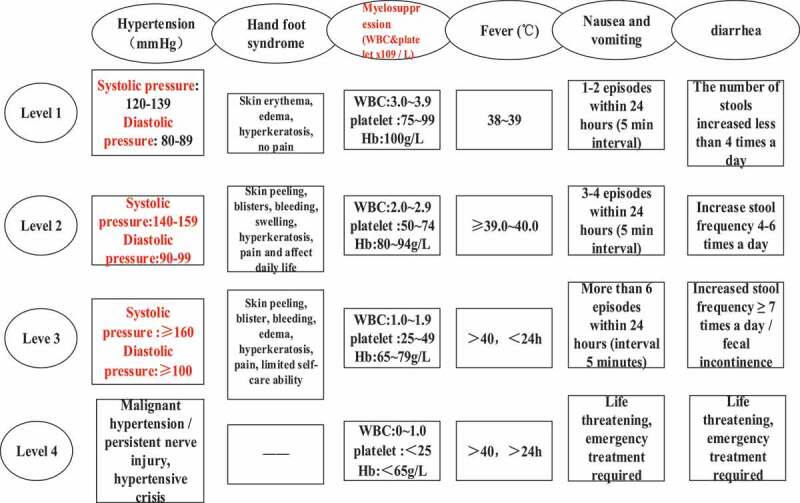
National cancer institute general toxicity standards

II. The evaluation criteria of adverse reactions were divided into levels one to four according to the National Cancer Institute General Toxicity Standard (NCI-CTC4.0). The specific grading standards were shown in Figure 3.

### 1.5. Statistical method

SPSS22.0 statistical software was used to analyze the data. Measurement data conforming to normal distribution were expressed as mean ± standard deviation (‾*X±s*), with two decimal places reserved. Pair t-test was used for intra-group differences, and independent sample t-test was used for inter-group differences. Quantitative data conforming to skewness distribution were represented by median (range), and Wilcixon rank sum test was used for differences between two groups. Enumeration data were expressed as percentage (%), with one decimal place reserved. Continuity correction of ×2 test or X2 test was used for differences between two groups. *P* value retained three decimal places, and *P* < 0.05 was considered statistically significant.

## 2. Results

In this study, it aimed to investigate the application value of TACE combined with targeted nanoparticle delivery of SFB system in the treatment of hepatocellular carcinoma with microvascular invasion. In order to further reflect the good application prospect of nanotechnology in the medical field, Ab-SFB-NP drugs were prepared and the therapeutic effect was compared with conventional drugs. The study results were as follows.

### 2.1. Characterization measurements

Through the measurement results of nuclear magnetic resonance analyzer, the characteristic peak of P123-Mal at 3.24 ppm and 3.58 ppm was obtained. The characteristic peak of -CH2 proton in the polyoxyethylene group of TPGS at 3.67 ppm demonstrated that P123 and TPGS-PCL were successful when combined. The average particle size of Ab-SFB-NP was 120.0 nm and the polymer dispersity index [PDI] = 0.18, indicating that the micellar particle size distribution was narrow and the nanoparticles were relatively homogeneous. The drug loading of Ab-SFB-NPs reached 10.3% by the detection of drug loading. These results suggest that Ab-SFB-NP was successfully prepared.

### 2.2. Differences of the general conditions of the two groups of patients

**Figure 4.**
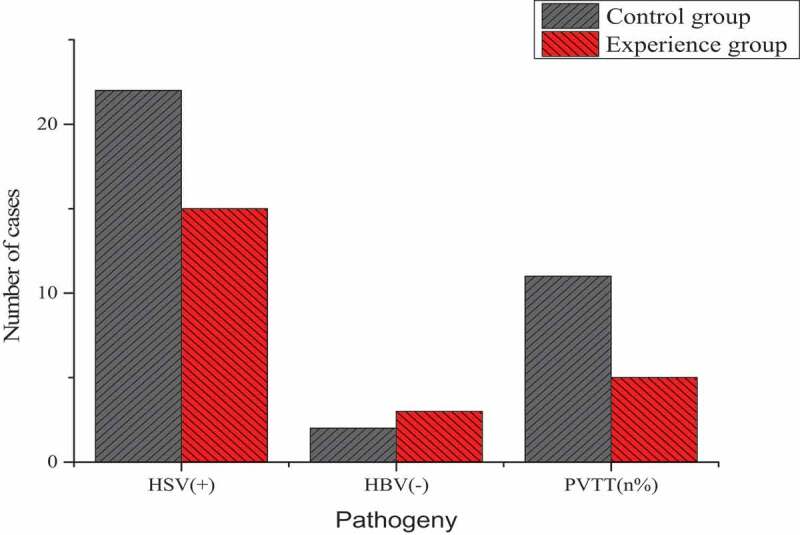
Differences of the causes of the two groups of patients

The age, gender, etiology, ECOG score, BCLC stage, and Child-Pugh level of patients in the two groups were compared as shown in Table 2 and Figure 4. The age of the experimental group was 54.22 ± 6.00 years old, and that of the control group was 55.32 ± 7.12 years old, and these differences were not statistically significant (*P* > 0.05). There were no statistically significant differences in gender, etiology, ECOG score, BCLC stage, and Child-Pugh level (*P* > 0.05).

**Table 2.** Differences of gender, ECOG score, BCLC staging, and Child-Pugh grading between the two groups

	Gender		ECOG scoring		BCLC stage		Child-Pugh level	
	Men	Women	0–1	2	B	C	A	B
The control group	14	4	8	10	9	9	14	4
The experimental group	21	3	11	13	10	12	17	7

### 2.3. Differences of the test results of the two groups of patients before treatment

**Figure 5.**
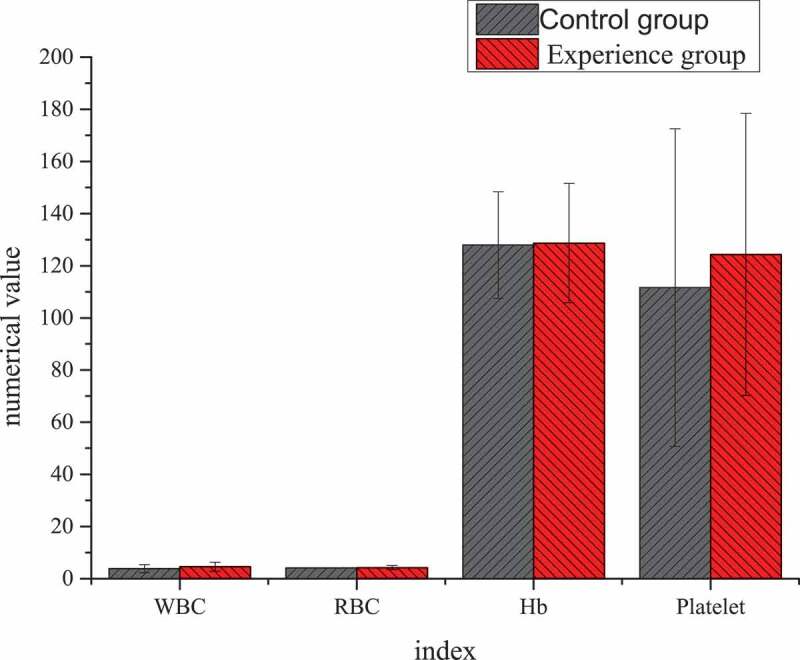
Differences of blood routine before TACE treatment between the two groups of patients. (Unit: white blood cell (WBC): ×10^9^/L; red blood cell (RBC): ×10^9^/L; Hemoglobin (Hb): g/L; Platelet: ×10^9^/L)

**Figure 6.**
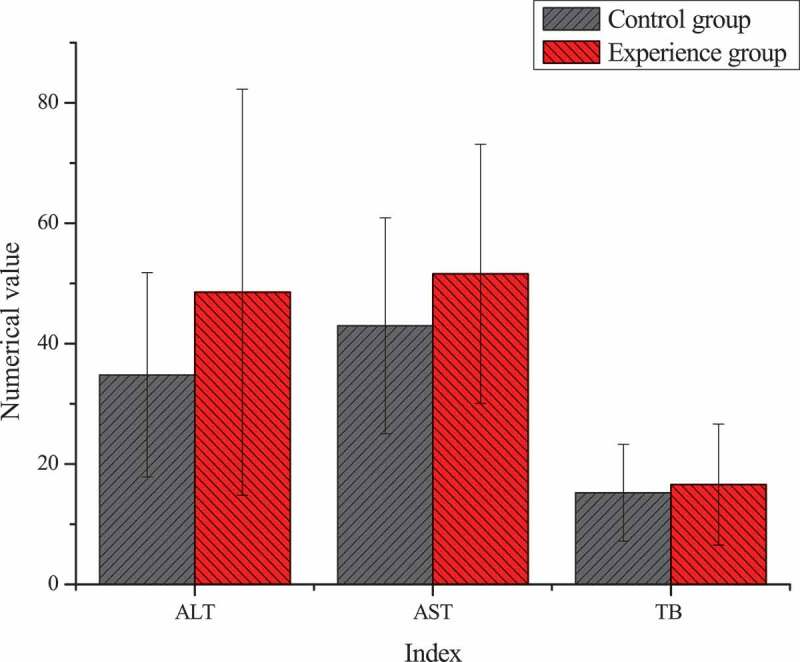
Differences of liver function between the two groups of patients before TACE treatment

The differences result of blood routine and liver function of patients in the two groups before TACE treatment were shown in Figures 5 and 6. Through statistical comparative analysis of the data of the two groups, it showed that the differences of preoperative blood routine and liver function between two groups of patients was not statistically significant (*P* > 0.05).

Unit: alanine aminotransferase, ALT, U/L; aspartate aminotransferase, AST, U/L; total bilirubin, TB, μmol/L

### 2.4. Differences of the test results of the two groups of patients after treatment

**Figure 7.**
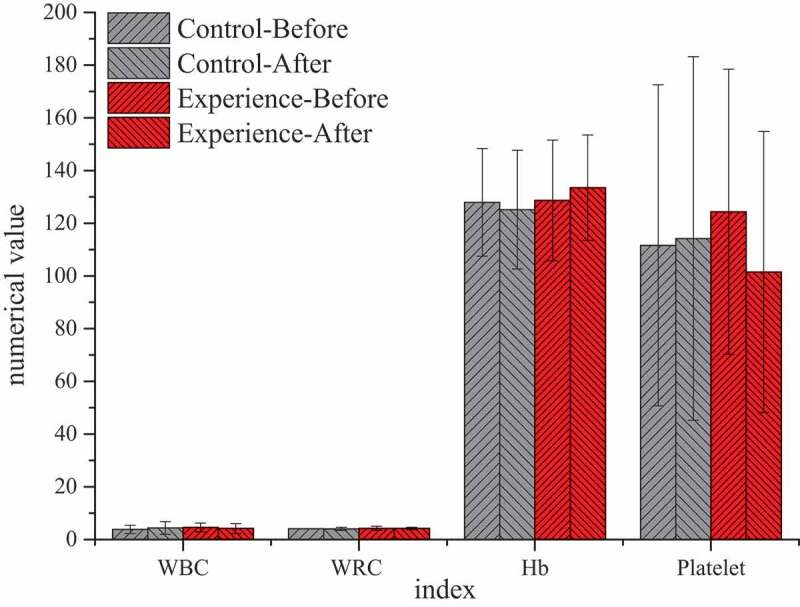
Comparison of blood routine between the two groups before TACE and after 3 months of treatment

**Figure 8.**
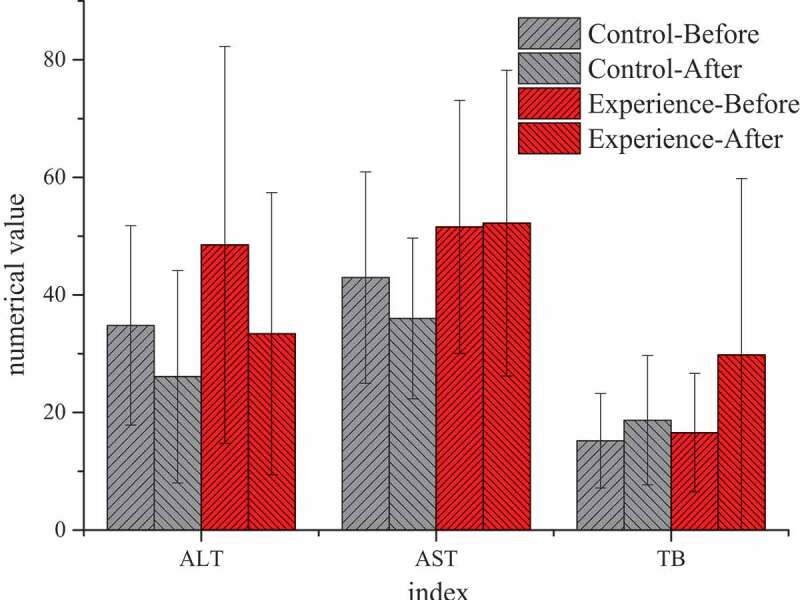
Comparison of liver function between the two groups before TACE and after 3 months of treatment

By comparing the test results of the two groups before TACE and at 1 and 3 months after treatment, it was found that there was no significant statistical significance in all indicators at 1 month after treatment (*P* > 0.05), without significant study effect. Therefore, all examination indicators were compared and analyzed before treatment and at 3 months after treatment. Figures 7 and 8 showed that there were significant differences in blood routine, liver function, and other laboratory indicators before and after treatment between the two groups. After 3 months of treatment, AST (52.21 ± 26.01) U/L in the experimental group was significantly higher than that (36.00 ± 14.00) U/L in the control group, with statistical significance (*P* < 0.05). There was no significant statistical significance in the results of other blood routine, ALT, TB, and other indicators (*P* > 0.05).

(Unit: WBC: ×10^9^/L; RBC: ×10^9^/L; Hb: g/L; Platelet: ×10^9^/L)

(Unit: ATL: U/L; AST: U/L; TB: μmol/L)

### 2.5. Differences of the therapeutic effect of the two groups of patients

Based on the enhanced CT and MRI detections one month and three months after TACE treatment, the treatment conditions of the two groups of patients were evaluated according to the evaluation criteria of mRECIST. After statistical analysis, it was found that in the experimental group under the combined treatment of TACE and Ab-SFB-NP nano-drug delivery system one month after treatment, there were six patients with CR, five patients with PR, one patient with PD, and six patients with SD, and the disease control rate (DCR) was 94.4%. In the control group treated with TACE and non-Ab-SFB-NP nano-drug delivery system, there were seven patients with CR, five patients with PR, seven patients with PD, and five patients with SD, and the DCR was 70.8%. The difference was not statistically significant (*P* > 0.05).

**Figure 9.**
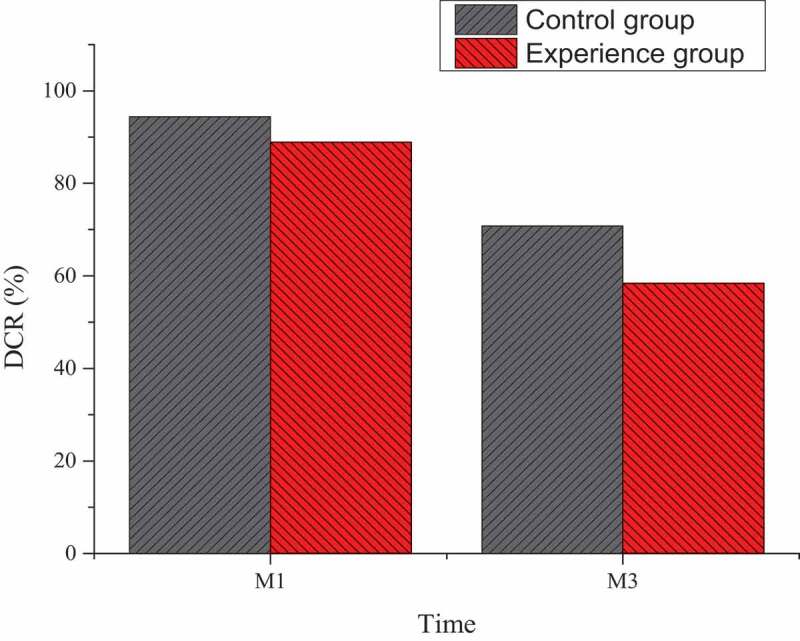
Differences of CDRs between the two groups at one month and three months after TACE treatment. (M1: one month, M3: three months)

Three months after treatment, there were four patients with CR, seven patients with PR, two patients with PD, and five patients with SD in the experimental group, and the DCR was 88.9%. In the control group treated with TACE and non-Ab-SFB-NP nano-drug delivery system, there were two patients with CR, seven patients with PR, ten patients with PD, and five patients with SD, and DCR was 58.4%. The difference was statistically significant (*P* < 0.05), as shown in Figure 9.

### 2.6. Adverse reactions after treatment in the two groups

The adverse reactions here included post-embolism syndrome and toxicity after adoption of SFB. Post-embolization syndrome mainly included abdominal pain, fever, nausea, and vomiting. Hand-foot syndrome, diarrhea, and bleeding were mainly SFB-related toxic reactions. In this experiment, there were 10 patients with abdominal pain, 14 patients with fever, and 7 patients with nausea and vomiting in the control group. Moreover, there were five patients with abdominal pain, seven patients with fever, and five patients with nausea and vomiting in the experimental group. After differences, there was no significant statistical significance in the differences of embolism syndrome between the two groups after treatment (*P* > 0.05). For the toxic reaction of SFB using the Ab-SFB-NP nano-drug delivery system, there were three patients with hand-foot syndrome, one patient with diarrhea, and one patient with bone marrow suppression, and the toxic reactions occurred mainly between level one to level two, and there was no occurrence above level three. After symptomatic treatment, the toxicity was effectively controlled and there was no death case. For the toxic effects of TACE and non-Ab-SFB-NP nano-drug delivery system treatment, there were five patients with hand-foot syndrome, five patients with diarrhea, and three patients with myelosuppression, and the toxic reactions occurred mainly between level two and level three, and one case occurred in level four. However, the toxicity was effectively controlled after symptomatic treatment, and no death cases occurred. The difference was statistically significant (*P* < 0.05), as shown in Table 3.

**Table 3.** Adverse reactions after treatment in the two groups

Grouping	Post-embolism syndrome			Toxicity reactions of SFB		
	Stomach ache	Fever	Nausea and vomiting	Hand-foot syndrome	Diarrhea	Bone marrow suppression
The control group	10	14	7	5	5	3
The experimental group	5	7	5	3	1	1

## 3. Discission

Because the process of liver cancer from the onset to the symptoms was very secretive, it was meaningless for most patients to undergo surgery when they were diagnosed. TACE, a non-surgical treatment method for liver cancer was recognized, but there were some inevitable risks in this treatment method [20]. The research found that a meta-analysis [21] included 11 studies, which were divided into adjuvant TACE group after liver cancer operation and surgical resection group alone. The results showed that compared with the surgical resection group alone, adjuvant TACE can prolong the disease-free survival and OS of patients with liver cancer and MVI, so it can be recommended for specific patients with liver cancer and MVI. Another study found that the average survival time of patients with primary liver cancer treated with TACE for one, two, three, and five years was 70.3%, 51.8%, 40.4%, and 32.4%, respectively, and confirmed that TACE can prolong the survival time of patients with advanced liver cancer [22]. However, some studies found that TACE treatment alone has the shortcomings of failing to eliminate residual cancer foci, stimulating the proliferation of residual cancer cells, vascular endothelial cells, and activating hepatitis virus replication, etc., and repeated TACE treatment may damage liver cells and aggravate the risk of cirrhosis [23]. Therefore, in this research, TACE therapy was combined with AB-SFB-NP nano drug delivery system for treatment. The research found that when HCC occurred, the activated Raf kinase activity was increased and caused it to overreact. In the development of HCC disease, the c-Raf/MEK/ERK signaling pathway played a very important role [24]. Tang et al. (2013) [25] reported that SFB could inhibit Raf kinase to block the MEK/ERK signaling pathway and reduce the level of cyclin D to limit the proliferation of PLC/PRF/5 and HepG2 HCC cells. In addition, SFB can also reduce the phosphorylation level of E IF4E and the expression of anti-apoptotic protein Mcl-1 by inhibiting the Raf/MEK/ERK signaling pathway [26]. However, SFB was administered orally. After the drug was absorbed through the intestinal mucosa, it first entered the liver through the hepatic portal vein. The level of the drug entered the blood circulatory system was easily affected by the pH value, the thickness and solubility of drug coating materials, the pathological changes in the digestive tract, and eating habits and other factors of ‘the homogeneous reservoir drug storeroom or airtight room’ [27]. Thus, the release property, bioavailability, and stability of the drug were affected. After continuous research and exploration, a new type of SFB nanoparticle AB-SFB-NP system for the targeted therapy of liver cancer has been developed. It proved that it had stable stability and can effectively release the anticancer drug SFB [28]. In this research, the target molecule of the AB-SFB-NP system was established with heparin sulfate proteoglycan phosphatidylinositol proteoglycan-3 (GPC3) as the research object. GPC3 showed to be overexpressed when HCC occurred, and it can also cause tumor cells to proliferate and metastasize through the Wnt signaling pathway, but it was expressed in normal liver tissues and other tissues. GPC3 can become the target molecule of HCC, and it can also induce the ADCC response to play an antitumor role by using the blocking antibody HGC33 [29]. As a glycoprotein that can penetrate cell membranes, GPC3 had a specific expression of nearly 80% for HCC [30,31]. Yao et al. (2013) [32] used immunohistochemical method to detect GPC3 in both HCC and non-hepatocellular tumors. The results of the research showed that GPC3 was widely positively distributed in the cytoplasm and cell membrane of liver cancer cells. In well-differentiated HCC tissues, the degree of positive expression of GPC3 in liver cancer cells was significantly higher than that of non-tumor cells around the lesion, and GPC3 was not expressed in the pathological tissues of focal nodular hyperplasia by immunohistochemical staining.

From the above analysis and discussion, TACE combined with AB-SFB-NP nano drug delivery system obviously had a better effect than TACE alone, and it also proved that the research of targeted molecular drug delivery system had its unique advantages for the treatment of liver cancer patients. The selective targeting of AB-SFB-NP to liver tumor tissue may offer promising strategies to reduce adverse effects of SFB and may help increase the treatment effect of SFB.

## 4. Conclusion

The results of this research were summarized as follows: (1) after three months of TACE, the control rate of using the Ab-SFB-NP nano-delivery system to deliver SFB was significantly higher than that of direct oral administration, and it had higher adoption value. (2) The toxic reactions of taking SFB with Ab-SFB-NP nano-drug delivery system mainly included hand-foot syndrome, diarrhea, and bleeding, and the toxic reactions occurred mainly between level one to level two, and there was no occurrence above level three. After symptomatic treatment, the toxicity was effectively controlled, there was no death case and SFB administered with the AB-SFB-NP nano-delivery system had high security. This study is relatively more detailed in the analysis and comparison of the grouping of patients, so that the results of the study are more accurate and meaningful. In a word, it can be imagined that this polymer nanoparticle could become a new nanomedicine platform for liver therapy, patients with advanced liver cancer who can’t be treated with surgery were brought to the hope of survival.
